# The effect of community health nurse-led multi-faceted group-based frailty prevention program for older adults: a multi-site pretest-posttest design

**DOI:** 10.1186/s12912-025-03372-7

**Published:** 2025-07-01

**Authors:** Gyeonga Kang, Hyungjoo Ji, Ju Young Yoon

**Affiliations:** 1 College of Nursing, Seoul National University, Seoul, Republic of Korea; 2 Nursing Administration Department, Seoul City Children’s Hospital, Seoul, Republic of Korea; 3 Research Institute of Nursing Science, Seoul National University, Seoul, Republic of Korea

**Keywords:** Older adults, Functional capacity, Frailty, Nurse-led, Group intervention

## Abstract

**Background:**

The number of vulnerable older adults is expected to continue to increase, highlighting the urgent need to develop comprehensive social care measures to address frail older adults’ needs. The objective of this study is to examine the effects of a community health nurse-led, multi-faceted, group-based frailty prevention program on functional capacity, frailty, and metabolic health among community-dwelling older adults.

**Methods:**

This quasi-experimental study uses a single-group, pretest-posttest design. Community dwelling older adults (≥ 65 years) were recruited from 13 counties using convenience sampling (*N* = 92). The 8-week program consisting of health education and exercise sessions was conducted by two per community health nurses with groups of 5 to 10 participants per county.

**Results:**

This study included 92 participants (mean age: 76.98 ± 5.43); 89.13% female. The intervention resulted in significant improvements in functional capacity, as measured by the TUG test time and grip strength (categorical) (t = 7.47, 95% CI [1.21, 2.08], *p*-value < 0.001; χ²=22.51, *p*-value < 0.001). Frailty showed significant improvements in both continuous (t = 7.17, 95% CI [1.34, 2.24], *p*-value < 0.001) and categorical indicators (χ²=29.71, *p*-value < 0.001). Among metabolic health indicators, only blood sugar levels showed significant improvement (χ²=8.95, *p*-value < 0.001).

**Conclusions:**

Although the absence of a control group limits the ability to draw direct causal inferences, improvements in health-related outcomes were observed to be associated with the intervention. Community health nurse-led, multi-faceted group interventions that are implemented based on a high understanding of county resources and participants may be an effective approach for developing health promotion programs for vulnerable older adults in community settings.

**Clinical Trial Number:**

Not applicable.

**Supplementary Information:**

The online version contains supplementary material available at 10.1186/s12912-025-03372-7.

## Background

The global population of older adults is increasing, with South Korea’s entry into a super-aged society accelerated to 2025. This trend is driven by longer life expectancy and the declining birth rate [1, 2]. Recent shifts in perceptions regarding elder care responsibilities emphasizing the accountability of the government and older adults, underscores the significance of community-based care for older adults [3]. Consequently, maintaining older adults’ physical and mental functions to delay their transition into long-term care is crucial not only for reducing healthcare costs but also for promoting healthy aging [3, 4].

Healthy aging and frailty exist on a continuum in the aging process. Research has shown that frailty can be reversed and improved [5]. It is recognized as a multidimensional concept encompassing physical, psychological, and social functional losses, with increasing vulnerability to adverse health outcomes such as disability, hospitalization, and death [4, 6]. Ultimately, improving older adults’ frailty is associated with maintaining functional independence and enhancing the quality of life, rather than simply extending their lifespan [7].

The multidimensional character of frailty necessitates comprehensive management [4, 6]. Previous studies have reported the efficacy of multi-faceted interventions to prevent frailty, comprising two or more components such as physical activity and educational approaches [8–11]. In particular, participation in diverse activities is recommended as the ultimate frailty prevention strategy for older adults [11]. Thus, multi-faceted interventions typically include various types of activities such as exercise, health education, and cognitive training [8, 9].

In South Korea, community health nurses are assigned at the county level, with an administrative system to help identify and provide care for vulnerable individuals [12]. Community health nurses interact with residents in their assigned areas and directly assess their health needs [13, 14]. Moreover, from the perspective of caring for transitional patients from clinical to community settings, community health nurses play a crucial role in the rehabilitation process due to their intimate understanding of the local community system [15]. Fundamentally, community health nurses can recruit vulnerable individuals and provide the required interventions using local resources. This approach makes it highly feasible to implement community health nurse-led frailty prevention programs in the community [16].

The number of vulnerable older adults is expected to continue to increase due to the growing prevalence of older adult couples and older adults living alone. Thus, it is urgent to develop comprehensive social care measures to address the needs of these community dwelling adults [1, 2]. Previous studies have demonstrated the effectiveness of nurse-led programs [10, 13]; however, insufficient community-based research has been conducted on such programs [17]. Therefore, this study aims to assess the impact of a community health nurse-led, multi-faceted, group-based frailty prevention program to address frailty among community-dwelling older adults aged 65 years or older.

## Methods

### Study design

This quasi-experimental study used a single-group pretest-posttest design to evaluate the effects of a community health nurse-led, multi-faceted, group-based intervention on functional capacity (TUG test, grip strength), frailty, and metabolic health indicators (BMI, blood pressure, blood sugar level) among older community-dwelling adults.

### Participants

This study was conducted in one large district in Seoul, encompassing 13 counties. Two community health nurses were assigned to each county and recruited adults aged 65 years or older using convenience sampling. In each area managed by the community health nurses, older adults who were suitable for study participation were introduced to the program, and their willingness to participate was confirmed. Among those who agreed to participate in this study, screening tests were performed on subjects who met the following criteria: (1) participants who could communicate in Korean and understand the purpose of this study and (2) older adults who had no significant difficulty performing physical activities and no severe cognitive impairment. Those who had been diagnosed with cognitive impairment by personnel in medical institutions such as hospitals were not eligible to participate. Researchers and community health nurses explained the purpose and methods of this study face-to-face, and all participants provided written informed consent. During data collection for this study, community health nurses assisted with the face-to-face completion of questionnaires to enhance data accuracy and minimize incomplete responses.

**Fig. 1:**
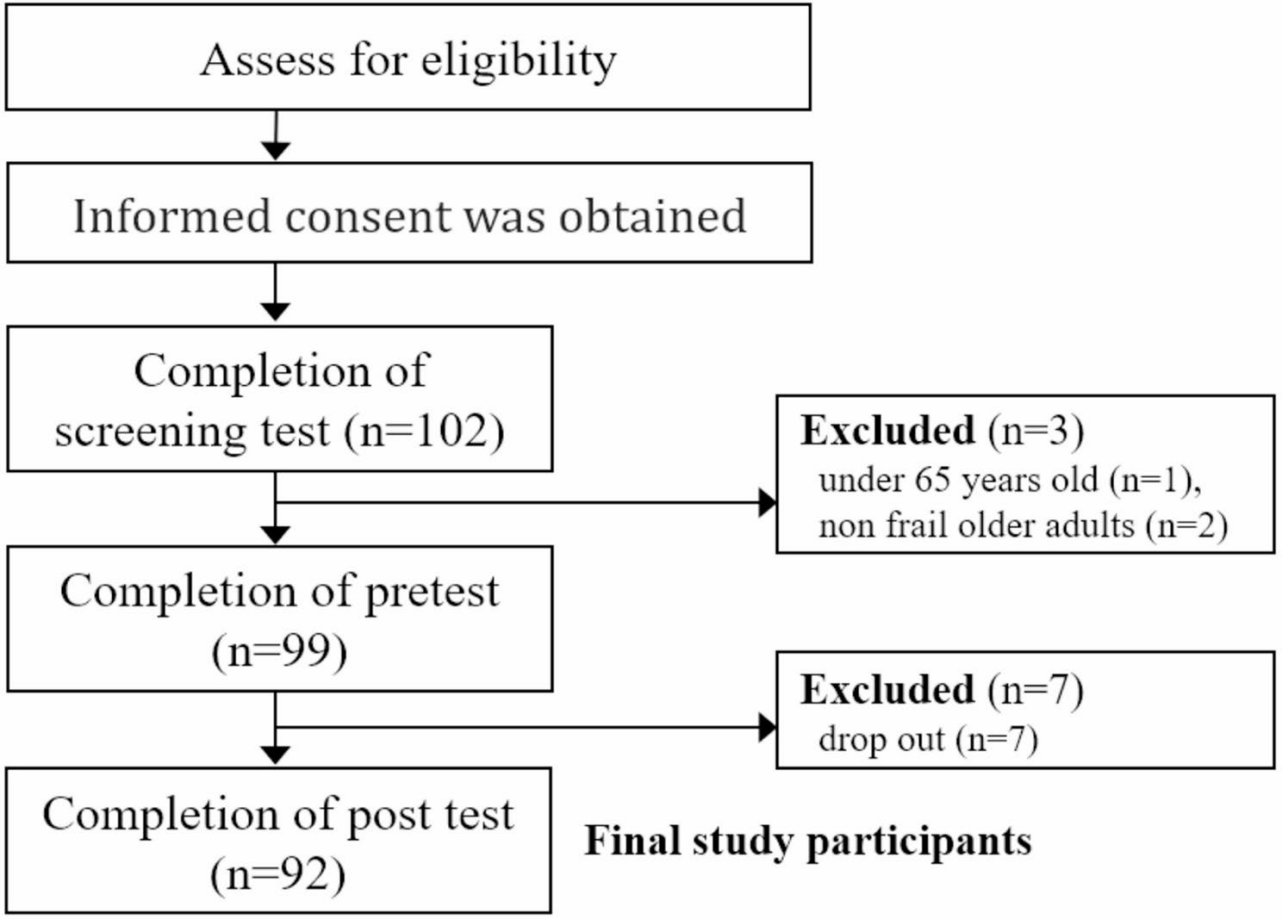
Flow chart for participant recruitment

The G*power 3.1.2 program was used to calculate the number of participants required for the study. The effect size was set at 0.3, which is a normal level. We also set a significance level of 0.05 and a power of 0.8 to calculate the sample size using a single group means comparison. The result was 90. We planned to recruit 157 participants considering that the dropout rate is typically 45%, but only 102 participants from 13 counties were recruited. The program was conducted by an average of two community health nurses who placed participants in groups of 5 to 10 older adults per administrative district based on the participants’ addresses (Supplementary Table 1). Among the 102 older adults who participated in the screening test, one person under 65 years old and two non-frail older adults (frail score less than 4) in the initial assessment were excluded. The pretest was performed on the remaining individuals. Seven participants withdrew from the study due to surgery or personal reasons and did not complete the post-survey; therefore, they were excluded from the final analysis. Furthermore, there was no missing data among participants who completed both the pre-survey and post-survey. Finally, 92 participants were selected as participants in this study and completed the 8-week program and post-survey. This number exceeded the minimum requirement of 90 participants for analysis (Fig. 1).

### Intervention program: community health Nurse-led, Multi-faceted, Group-based, frailty prevention program

The Community Health Nurse-led, Multi-faceted, Group-based, Frailty Prevention Program was designed to enable community health nurses to plan the program that included health education session and exercise session for older adults. The health education sessions covered topics such as nutrition education, oral health education, dementia prevention (e.g., understanding dementia screening tests, cognitive programs for dementia prevention), chronic disease management education, and mental health management (e.g., horticultural therapy, storytelling). The community health nurses selected six content areas from the topics to include in the program they would conduct in their respective counties.

One of the key areas was the exercise session. Each session included a portion of The Standardized Indoor Exercise Program developed by the Ministry of Health and Welfare (MOHW) and the Korea Health Promotion Institute (KHPI) to prevent sarcopenia and falls in older adults. It incorporated exercises aimed at improving strength, balance, flexibility, and cardiorespiratory endurance. This program was led by physical activity specialists or health exercise managers who are responsible for physical activity education in public health centers. Details on The Standardized Indoor Exercise Program referenced in this study are available on the 2023 KHPI website under the name Elderly Strength and Balance Exercise Completion Program Manual. It is registered under the Public Nuri Type 4 guidelines with free access (https://www.khepi.or.kr/).

The Multi-faceted, Group-based, Frailty Prevention Program in this study was conducted by trained community health nurses to ensure consistency in program delivery. The nurses were given printed materials developed by KHPI in advance. Then, the community health nurses were trained on the standardized indoor exercise program in a 3-hour in-person workshop focusing on the exercise guide. In addition, the survey used in this study was developed for this study (Supplementary file 1). The nurses were trained to use survey tools and measurement methods through an interactive Q&A. The community health nurses planned the 8-week program for their respective counties based on the unique characteristics of the participants they intended to recruit. They tailored both the health education content and exercise contents accordingly (Supplementary Table 2). Community health nurses were instructed to consult the research team at any time should questions arise regarding the intervention.

The intervention consisted of 12 h over 8 weeks, with one 90-minute session per week. Each weekly session was divided into a 30-minute health education session and a 60-minute exercise session. At the beginning of each session, the community health nurses introduced the program and facilitated ice-breaking activities. In weeks 1 and 8, the education session was replaced with a pretest and posttest to evaluate program effectiveness. In addition, a program satisfaction survey was conducted in week 8.

The program was conducted in groups of 5–10 participants. The health education sessions were primarily conducted by community health nurses for each county. However, some sessions were delivered by invited experts. The exercise sessions were conducted by two community health nurses: one demonstrated the movements while the other corrected participants’ postures. Throughout the sessions, the community health nurses encouraged the participants with motivational remarks. After two weeks of exercise sessions, the number of repetitions was adjusted according to participants’ abilities. The supplies participants needed to perform the exercises (e.g., water bottles) were prepared by the community health nurses. To promote consistent participation, community health nurses contacted participants via phone a day before each session to provide schedule reminders and encouragement. To prioritize safety, all sessions began with an assessment of participants’ physical conditions (e.g., joint pain, blood pressure). Water was also available and encouraged for all sessions to ensure hydration. Participants were closely monitored by the community health nurses to prevent injuries, and chairs were provided so participants could take breaks at any time (Table 1, Supplementary Table 3).

**Table 1:** Content of the intervention

Component (time)	Content	Teaching method
Health education session (30 min)	∙ Nutrition (e.g., low-salt, high-protein diet) ∙ Oral health (e.g., brushing teeth properly), ∙ Chronic disease management (e.g., high blood pressure and diabetes management methods)	Face-to-face group activities / Lecture / Handout materials
	∙ Mental health management (e.g., gardening activities, reading fairy tales) ∙ Dementia prevention (e.g., understanding dementia screening tests, cognitive program for dementia prevention.)	Face-to-face group activities / Hands-on practice / Taught by invited experts
Exercise session (60 min)	∙ Warm up (10 min): proper walking posture, standing shoulder stretch, standing side stretch ∙ Main exercise (40 min): seated water bottle bicep curls, seated water bottle lateral raises, seated water bottle triceps extensions, seated water bottle front raises, sit-to-stand from chair, standing knee lifts, standing knee bends, standing side leg raises ∙ Wrap up (10 min): marching in place, 6-direction neck stretch, shoulder stretch, chest stretch, back and rear shoulder stretch, torso rotation, seated toe touch, seated knee to chest, calf stretch	Face-to-face group exercise program / Instructor-led demonstration / Participant imitation of exercises / Handout materials

Note. Community health nurses selected six topics for health education sessions spanning six weeks. Either the nurses or invited experts provided health education for specific topics. Additionally, the nurses identified and incorporated various exercise components that were suitable for participants in each county based on the content presented in the exercise sessions.

### Measures

#### General characteristics

The following general characteristics of the participants were identified: gender (Male vs. Female), age (years, 65–74 vs. ≥75), living with a spouse (Yes vs. No), living alone (Yes vs. No), educational level (≤ 6 vs. 6< ), and current working status (Yes vs. No).

#### Functional capacity: TUG, grip strength

The Timed Up-and-Go (TUG) test assesses balance and gait function in older adults [18]. For the test, participants were asked to rise from a seated position in a chair, walk 3 m in a straight line, turn around, and sit back down. The time it took from getting up from the seat to sitting down again was measured in seconds.

Grip strength can be used as an indicator to assess the overall functional capacity of participants and is associated with various health outcomes in older adults [19]. It was measured using a hand dynamometer (model: CAMRY EH101). The highest grip strength was determined by measuring both left and right hands twice. Grip strength has been identified as both a continuous indicator and a categorical indicator (low grip strength: <28 kg for males, < 18 kg for females vs. normal grip strength) [20].

#### Frailty

Frailty was measured using the Elderly Health Interview Questionnaire in the Home Visiting Health Care Service, which is part of the Community Integrated Health Promotion Project [21]. This instrument consists of 28 items: 5 items on activities of daily living, 3 items on mobility, 2 items on falls, 5 items on nutrition (including 3 items on oral health), 2 items on social health, 3 items on cognitive function, 5 items on depression, 1 item on chronic diseases, 1 item on sensory function related to vision and hearing, and 1 item on gait function. Each item is scored as “Yes” 1 point or “No” 0 points, except for items 1–8, 16, and 19, which are positive items and scored as 1 point when answered “No.” The items on chronic diseases, sensory function, and gait function could receive up to 2 points. Total scores range from 0 to 31 points, with higher scores indicating lower functional status and severe frail status. Scores of 4–12 points are classified as pre-frailty, and 13 points or higher as frailty. In the previous study using this instrument, Cronbach’s α was.81 [22].

#### Metabolic health indicators: BMI, blood pressure, blood glucose level

Body mass index (BMI), blood pressure, and blood glucose are key components of metabolic syndrome, which is a cluster of conditions that increase the risk of heart disease, stroke, and diabetes [23, 24]. Previous studies on the health outcomes of older people have identified BMI as both a continuous and categorical variable (< 25 [not obese] vs. ≥25 [obese]) [25]. Blood pressure, including systolic and diastolic blood pressure, was measured using a blood pressure monitor (model: Inbody bp170) and identified as both a continuous and categorical variable (SBP: < 130 vs. ≥130; DBP: < 80 vs. ≥80) [26]. Blood glucose was measured using a portable blood glucose meter (model: ACCU-CHEK PERFORMA MODEL NC), where a lancet momentarily punctures the skin to apply a drop of blood to the test strip for glucose measurement. It was identified as both a continuous and categorical variable (< 140 vs. ≥140), considering the difference in health outcomes for participants around 140 mg/dl [27].

### Data collection

Data collection for this study was conducted from April to November 2024 in 13 counties in Seoul under a public health center that agreed to participate in the study. With permission from the public health center, the community health nurses in each center were informed about the research purpose and were asked if they were willing to cooperate. Nurses identified older adults living in the community who were then informed about the program’s purpose and procedures by the community health nurses and voluntarily agreed to participate. Physical measurements and surveys consisting of 42 items were conducted by the community health nurses. The assessments included general characteristics (6 items), physical measurements (8 items), and frailty (28 items). Satisfaction (6 items) was only assessed in the posttest). The time required for physical measurements and surveys was approximately 20 min.

### Statistical analysis

The collected data were statistically analyzed using SPSS version 27.0. (IBM, New York, United States). First, participants’ general characteristics were examined by descriptive statistics. Second, the effectiveness of the program at two points (pre-intervention, post-intervention) was analyzed using a t-test and chi-square test. Furthermore, bootstrapped paired t-tests and non-parametric Wilcoxon signed-rank tests were conducted to account for potential normality violations in continuous variables. The McNemar test was used to analyze some categorical variables. The statistical significance level was set at.05.

### Ethical considerations

This study was conducted per the Declaration of Helsinki. This study, including all experiments involving human participants, was conducted after obtaining approval from the institutional review board (IRB) of the university to which the researchers are affiliated (IRB No. 2404/004–011). Before commencing the study, the purpose and procedures were explained to the participants, and all participants provided written informed consent. When necessary, the consent form was provided by a legal guardian or an appropriate representative on behalf of the participant. We also gave participants detailed information regarding the protection of their rights and ensured data anonymity.

## Results

### General characteristics

The study included 92 participants, with 89.13% female and 10.87% male. The average age of the participants was 76.98 years; 68.48% of the participants were 85 years or older. The average years of education was 6.76 years. Over half (58.70%) of the participants had 6 years or less of education, considering that elementary school graduation requires 6 years. Among the participants, 67.39% were living alone, and 10% were currently working.

Regarding the metabolic health indicators in the pretest, 28.26% of the participants were identified as obese with a BMI of 25 kg/m2 or higher. Almost half (47.83%) of the participants had systolic blood pressure of 130 mmHg or higher, while 27.17% had diastolic blood pressure of 80 mmHg or higher. Additionally, 56.52% of the participants had a simplified blood glucose level of 140 mg/dl or higher (Table 2). A comparison between study completers and dropouts was conducted to assess potential attrition bias, revealing no significant differences in sociodemographic characteristics, metabolic health indicators, or functional capacity (Supplementary Table 4).

**Table 2:** Characteristics of the participants in baseline (*N* = 92)

Variables		*n* (%) or M ± SD
*Sociodemographic factors*		
Gender	Male	10 (10.87)
	Female	82 (89.13)
Age (continuous)		76.98 ± 5.43
Age (categorical)	65–74	29 (31.52)
	≧ 85	63 (68.48)
Spouse cohabitation status	No	61 (66.30)
	Yes	31 (33.70)
Live alone	No	30 (32.61)
	Yes	62 (67.39)
Education level (continuous)		6.76 ± 3.90
Education level (categorical)	≦ 6 years	54 (58.70)
	> 6 years	38 (41.30)
Current working status	No	82 (89.13)
	Yes	10 (10.87)
*Metabolic health indicators*		
BMI		23.09 ± 3.45
	< 25 kg/m^2^	66 (71.74)
	≧ 25 kg/m^2^	26 (28.26)
SBP (mmHg)		129.21 ± 20.87
	< 130	48 (52.17)
	≧ 130	44 (47.83)
DBP (mmHg)		73.01 ± 11.69
	< 80	67 (72.83)
	≧ 80	25 (27.17)
BST (mg/dl)		159.72 ± 58.30
	< 140	40 (43.48)
	≧ 140	52 (56.52)
*Functional capacity*		
Grip strength (kg)		19.91 ± 4.92
Low grip strength (Male ≦ 28 kg, Female 18 kg)	Yes	40 (43.48)
	No	52 (56.52)
Frailty		8.32 ± 2.85
	Pre-frail (4–12)	86 (93.48)
	Frail ( ≧ 13)	6 (6.52)

Note. Means (M) ± SD for continuous variables and frequencies (n) with percentages (%) for categorical variables are presented.

### Comparison of functional capacity, frailty, and metabolic health in the pre- and post-intervention outcomes

A comparison of pre- and post-intervention outcomes revealed a significant improvement in frailty scores (t = 7.17, 95% CI [1.34, 2.24], *p*-value < 0.001), along with notable changes in categorical indicators (χ²=29.71, *p*-value < 0.001). In terms of functional capacity, significant improvements were observed in the TUG test time and grip strength (categorical) (t = 7.47, 95% CI [1.21, 2.08], *p*-value < 0.001; χ²=22.51, *p*-value < 0.001). Within the domain of metabolic health, a significant change was identified only in blood glucose levels (χ²=8.95, *p*-value < 0.001), as shown in Table 3. As presented in Supplementary Table 5, bootstrapped and non-parametric results were consistent across methods.

**Table 3:** Comparison of metabolic health indicators and functional capacity in pre-intervention (baseline) and post-intervention assessments of older adults (*N* = 92)

	*n* (%) or M ± SD					
Variables	Pre-intervention	Post-intervention	t or χ²	*p*-value	Cohen’s d	95%CI
*Metabolic health indicators*						
BMI (kg/m^2^)	23.09 ± 3.45	23.16 ± 3.41	-1.02	0.311	-0.11	[-0.19, 0.06]
< 25 kg/m^2^	66 (71.74)	65 (70.65)	78.01	1.000†		
≧ 25 kg/m^2^	26 (28.26)	27 (29.35)				
SBP (mmHg)	129.21 ± 20.87	127.09 ± 17.22	1.24	0.219	0.13	[-1.29, 5.52]
< 130	48 (52.17)	52 (56.52)	24.97	0.523†		
≧ 130	44 (47.83)	40 (43.48)				
DBP (mmHg)	73.01 ± 11.69	72.54 ± 10.76	0.43	0.665	0.05	[-1.67, 2.61]
< 80	67 (72.83)	71 (77.17)	26.93	0.453†		
≧ 80	25 (27.17)	21 (22.83)				
BST (mg/dl)	159.72 ± 58.3	139.28 ± 50.30	4.54	< 0.001	0.47	[11.50, 29.37]
< 140	40 (43.48)	63 (68.48)	8.95	< 0.001†		
≧ 140	52 (56.52)	29 (31.52)				
*Functional capacity*						
TUG TEST (sec)	9.65 ± 3.45	8.00 ± 3.07	7.47	< 0.001	0.78	[1.21, 2.08]
<8.5	46 (50.00)	65 (70.65)	23.21	< 0.001		
≧8.5	46 (50.00)	27 (29.35)				
Grip strength (kg)	19.91 ± 4.92	21.05 ± 5.18	-4.22	< 0.001	-0.44	[-1.68, -0.60]
Low grip strength Yes	40 (43.48)	20 (21.74)	22.51	< 0.001†		
No	52 (56.52)	72 (78.26)				
Frailty	8.32 ± 2.85	6.72 ± 2.67	7.17	< 0.001	0.82	[1.34, 2.24]
Non-frail (0–3)	0 (0)	11 (11.96)	29.71	0.004		
Pre-frail (4–12)	86 (93.48)	79 (85.87)				
Frail ( ≧ 13)	6 (6.52)	2 (2.17)				

Note. t-values for continuous variables and chi-square values for categorical variables are presented. Means (M) ± standard deviation (SD) for continuous variables and frequencies (n) with percentages (%) for categorical variables are presented. Low grip strength is defined as < 28 kg for males and < 18 kg for females. 95% CI is provided for the effect size (Cohen’s *d*) of continuous variables.

†Calculated using a McNemar test

### Comparison of specific frailty domains in the pre- and post-intervention

Comparing the changes in the specific domains of the frailty pre- and post-intervention, significant improvements were observed in the following areas: activities of daily living, mobility, social health, depressive symptoms, sensory function, and gait function (TUG test). Substantial changes were seen in gait function (t = 4.12, 95% CI [0.21, 0.64], *p*-value < 0.001), and depressive symptoms (t = 4.00, 95% CI [0.19, 0.55], *p*-value < 0.001). Additional details are presented in Supplementary Table 6.

### Satisfaction of study participants

At the end of the 8-week program, we surveyed the 92 participants to assess their satisfaction with the program using a 5-point Likert scale (0: very poor to 5: very good) across five areas: helpfulness in health management, satisfaction with operation, intention to participate in the future, willingness to recommend participation to others, and helpfulness in acquiring health information. The results for the items were 4.88 ± 0.39, 4.92 ± 0.34, 4.91 ± 0.28, 4.88 ± 0.39, and 4.89 ± 0.35, respectively, with the highest satisfaction in willingness to recommend participation to others. The overall satisfaction of the study participants was very high (24.48 ± 0.78 of the 25 total points).

The qualitative survey results on participant satisfaction revealed positive perspectives. Sample comments include, “The exercise was suitable for our age,” “It was good to learn how to exercise,” “It was nice to meet other people,” “It was good to get to know neighbors,” “It was good that nurses directly conducted the program,” “I was happy,” and “It was fun.” Suggestions for program improvement included, “I wish it lasted longer,” “It would be nice to add dance,” and “It would be nice to add crafting programs.”

### Post-program comments on program operation from the community health nurses

We also qualitatively surveyed community health nurses’ perspectives on effective program operation. Successful aspects of the program operation included the following perspectives: “Calling participants the day before the program to build rapport and encourage participation seemed to help with program operation,” “It was good to have a location with mirrors secured for the exercise program,” “When the program ran for more than 30 minutes, participants seemed to get bored, so we provided breaks by clapping hands or engaging in casual conversation. It seems important to provide time for refreshment during the program,” “Participants were very pleased when given small gifts.“, “The decision to have two-nurse teams at work in each county contributed positively to the successful implementation of the program.”

Improvement suggestions for the program operation included: “Locations without elevators were inconvenient for older adults to access,” “When there were large differences in education levels among participants, some found the educational content too easy and felt bored.”

## Discussion

This study examined the effects of an 8-week Community Health Nurse-led Multi-faceted Group-based Frailty Prevention Program on functional capacity, frailty, and metabolic health in community-dwelling older adults aged 65 and older using a single-group pre-post comparison design. The intervention demonstrated significant improvements in functional capacity, particularly in the TUG test and grip strength. A significant overall improvement in frailty was observed when examining changes in specific frailty domains, with notable improvements in gait function and depressive symptoms. Furthermore, participants showed significant improvements in blood glucose levels, a key indicator of metabolic health. Participant satisfaction with the program and community health nurses’ perspectives on effective program operation were also assessed, revealing positive feedback on the program’s content and nurse-led group management approach.

The participants in this study were identified by community health nurses as vulnerable older adults in pre-frail or frail conditions within their assigned counties. Regarding the demographic characteristics of the study participants, 89.13% were female. This aligns with previous research findings that generally show a higher prevalence of frailty among female older adults compared to male older adults [28]. Regarding education levels, 58.7% of the participants had an elementary school education or lower, indicating a lower education attainment. Compared to the approximate average of 80% of frail or very frail older adults nationwide who have an education level of elementary school or below [29], the education level of the participants in this study was relatively higher. This higher education level likely reflects the trend of decreasing illiteracy rates and increasing high school education rates among older adults in Seoul, South Korea [1]. Over 60% of the participants lived alone, implying that community health nurses effectively identified vulnerable individuals requiring community care.

Despite the intervention lasting only 8 weeks, significant improvements were observed in TUG test time and grip strength, which are indicators of functional capacity. The reduction in TUG test time reflects improvements in lower limb strength, balance ability, and walking speed [18]. Enhanced gait function can also expand overall activity range, potentially leading to improved independence and quality of life for older adults [8, 30]. Grip strength improvement reflects enhanced upper limb strength [31], which is also associated with improved independence and ability to perform daily activities [25]. Positive feedback from participants included comments such as “I learned exercise methods” and “The exercise level was easy to follow,” as well as appreciation for nurse-led instruction. The results of the intervention and satisfaction survey indicated that community health nurses effectively tailored the program to participants’ physical conditions, prioritizing manageable exercises. The nurses’ understanding of participants’ physical characteristics and appropriate selection of exercises likely contributed significantly to their improved functional capacity [13].

The intervention in this research aimed to improve frail conditions for healthy aging. A multi-faceted intervention was implemented based on the multidimensional concept of frailty [4–6], resulting in significant improvement in overall frailty scores. Two-thirds of the program consisted of exercise, which has been shown to be effective in improving overall frailty status. Similar to previous research [10, 25, 32], this study found that physical exercise was effective in improving the overall frail status of older adults. Multi-faceted interventions with a focus on physical exercise are expected to be effective in improving frailty among frail older adults. In addition, improvement in depressive symptoms and social health were also observed.

In this study, the intervention was conducted in small groups of 5 to 10 participants. Satisfaction surveys revealed positive feedback such as “It was good to get to know neighbors” and “It was nice to meet other people,” which aligns with previous research showing that group interventions can increase social interaction between participants [33]. Strengthening social networks through social interactions can also alleviate depressive symptoms and significantly contribute to overall life satisfaction in older adults [33, 34]. Considering that 67.39% of participants lived alone, the group-based approach of the program likely strengthened the effects of the program to improve depressive symptoms by promoting social interactions among participating older adults.

Regarding metabolic health indicators, significant improvements in blood glucose levels were observed. It is likely that exercise enhanced insulin sensitivity and glucose utilization [35]. However, no significant improvements in blood pressure or BMI were observed. Given that short-term weight fluctuations in older adults can be a risk for frailty [36], the absence of BMI changes may be considered appropriate. Blood pressure improvements also typically require sustained behavioral changes over extended periods, with evidence suggesting at least 24 months of intervention for meaningful outcomes [37, 38]. Thus, the 8-week duration in this study may have been insufficient to improve blood pressure among participants. Furthermore, in Korea, healthier food options are often relatively expensive [39], making it challenging for vulnerable older adults to purchase food for a healthier diet. Thus, active strategies such as providing food as well as education on blood pressure management should be implemented to address these barriers [37, 40, 41].

Community health nurses’ perspectives can be categorized into facilitating factors and barriers to operating health promotion programs for older adults. Facilitating factors included establishing rapport between community health nurses and participating older adults before the intervention, appropriately utilizing material resources (e.g., good locations for program operation, small gifts), and providing adequate rest time. These findings align with previous research indicating that simple phone-based interventions help seniors maintain adequate weight and physical function [42]. Conversely, disparities in participants’ education levels were identified as a barrier to delivering education sessions. The information that can be provided to older adults with lower education is inherently limited, and socioeconomic differences may exacerbate health equity gaps [43]. These disparities can be partially addressed by employing diverse educational methods, such as peer education, to address varying comprehension levels [44]. Enhancing facilitating factors and addressing barriers will improve program effectiveness.

In this study, an 8-week nurse-led program managed by professional nurses was fully implemented. Professional confidence among nurses is a critical factor for successfully operating community base health promotion programs. In addition to training, confidence can be acquired through experience [45]. Through the process of independently planning, implementing, and evaluating programs, nurses can enhance their own competencies and build confidence in their professional capabilities.

Despite the important results of this study, this study has some limitations. First, as a single-group pre- and post-intervention study without a control group, it is difficult to clearly identify the pure effect of the intervention. Second, although participants were sampled using convenience sampling from each county, focusing on only one district, Gangbuk-gu in Seoul, limits generalizability. Third, the intervention in this study was applied for only 8 weeks, making it difficult to assess long-term effects and the influence of seasonal changes. Therefore, further long-term studies with stronger research designs, such as randomized controlled trials (RCTs), and a larger number of participants from diverse regions are needed, with efforts to implement blinded outcome assessments to ensure more objective data collection.

## Conclusion

Although the absence of a control group limits the ability to draw direct causal inferences, the 8-week intervention program in this study was associated with improvements in functional capacity, frailty, and metabolic health. By leading this intervention, community health nurses delivered tailored exercise and educational interventions considering the characteristics of older adults in each county, and utilizing appropriate community resources to achieve more effective results. Delivering the program in small groups is believed to have maximized the program’s effect on improving depressive symptoms by promoting social interaction among older adult neighbors. In conclusion, utilizing community health nurses to lead multi-faceted group interventions based on a deep understanding of county resources and participants, may be an effective approach for such programs. Further efforts should target the development of health promotion programs for vulnerable older adults in the community in the future.

## Electronic supplementary material

Below is the link to the electronic supplementary material.

Supplementary Material 1

Supplementary Material 2

## Data Availability

The datasets used and/or analyzed during the current study are available from the corresponding author upon reasonable request.

## Abbreviations

KHPI: Korea Health Promotion Institute
MOHW: Ministry of Health and Welfare
WHO: World Health Organization
