# Supplementary material for: The effect of community health nurse-led multi-faceted group-based frailty prevention program for older adults: a multi-site pretest-posttest design
Source: BMC Nurs. 2025 Jul 1;24:746. doi: 10.1186/s12912-025-03372-7 (PMC12211464; doi:10.1186/s12912-025-03372-7)
Supplement: Supplementary file 1 — Supplementary Material 1 [file 12912_2025_3372_MOESM1_ESM.docx]

**Satisfaction Survey – To be completed only after the 8th week of the program**

| Satisfaction Survey Content | | **Strongly Agree** | **Agree** | **Neutral** | **Disagree** | **Strongly Disagree** |
| --- | --- | --- | --- | --- | --- | --- |
| 1. Has the health group meeting been helpful for your health management? | |  |  |  |  |  |
| 2. | Are you overall satisfied with the operation of the health group meeting? |  |  |  |  |  |
| 2-1. | If you are dissatisfied, please write down the reason. |  | | | | |
| 3. Would you be willing to participate in future health group programs? | |  |  |  |  |  |
| 4. Would you recommend the health group meeting to others? | |  |  |  |  |  |
| 5. Has the health group meeting been helpful for acquiring health information? | |  |  |  |  |  |
| 6. If you have any suggestions for improving the program, please feel free to share. What aspects did you like, and what aspects do you feel could be improved? | | | | | | |
