# Supplementary material for: The effect of community health nurse-led multi-faceted group-based frailty prevention program for older adults: a multi-site pretest-posttest design
Source: BMC Nurs. 2025 Jul 1;24:746. doi: 10.1186/s12912-025-03372-7 (PMC12211464; doi:10.1186/s12912-025-03372-7)
Supplement: Supplementary file 2 — Supplementary Material 2 [file 12912_2025_3372_MOESM2_ESM.docx]

**Supplementary Table 1.** Participant ratio by county (N=92)

| **County** | **n (%)** |
| --- | --- |
| Total | 92 |
| Samyang-dong | 5 (5.43) |
| Mia-dong | 6 (6.52) |
| Songjung-dong | 5 (5.43) |
| Songcheon-dong | 8 (8.7) |
| Samgaksan-dong | 8 (8.7) |
| Beon 1-dong | 10 (10.87) |
| Beon 2-dong | 8 (8.7) |
| Beon 3-dong | 8 (8.7) |
| Suyu 1-dong | 9 (9.78) |
| Suyu 2-dong | 7 (7.61) |
| Suyu 3-dong | 6 (6.52) |
| Ui-dong | 6 (6.52) |
| Insu-dong | 6 (6.52) |
| **Note.** Dong is the smallest urban administrative unit with its own office and staff. | |

**Supplementary Table 2.** Community health nurse program operation plan sample of Suyu 1-dong

| **Week** | **Component** | **Time (min)** | **Contents** | **Instructor** |
| --- | --- | --- | --- | --- |
| 1 | Introduction | 10 | Program introduction and schedule overview | CHN (2) |
|  | Pretest | 30 | Health status assessment |  |
|  | Exercise session | 40 | Indoor exercise for the elderly |  |
|  | Closing | 10 | Wrap-up and next week’s schedule announcement |  |
| 2 | Introduction | 10 | Program introduction and schedule overview | CHN (2),  Dementia center staff (1) |
|  | Education session | 30 | Understanding dementia screening tests |  |
|  | Exercise session | 40 | Indoor exercise for the elderly |  |
|  | Closing | 10 | Wrap-up and next week’s schedule announcement |  |
| 3 | Introduction | 10 | Program introduction and schedule overview | CHN(2), Dementia center staff (1) |
|  | Education session | 30 | Cognitive program for dementia prevention |  |
|  | Exercise session | 40 | Indoor exercise for the elderly |  |
|  | Closing | 10 | Wrap-up and next week’s schedule announcement |  |
| 4 | Introduction | 10 | Program introduction and schedule overview | CHN (2) |
|  | Education session | 30 | Chronic Disease Management Education (Focused on hypertension and diabetes management) |  |
|  | Exercise session | 40 | Indoor exercise for the elderly |  |
|  | Closing | 10 | Wrap-up and next week’s schedule announcement |  |
| 5 | Introduction | 10 | Program introduction and schedule overview | CHN (2) |
|  | Education session | 30 | Sarcopenia Prevention and Nutrition Education |  |
|  | Exercise session | 40 | Indoor exercise for the elderly |  |
|  | Closing | 10 | Wrap-up and next week’s schedule announcement |  |
| 6 | Introduction | 10 | Program introduction and schedule overview | CHN (2) |
|  | Education session | 30 | Reading fairy tales |  |
|  | Exercise session | 40 | Indoor exercise for the elderly |  |
|  | Closing | 10 | Wrap-up and next week’s schedule announcement |  |
| 7 | Introduction | 10 | Program introduction and schedule overview | CHN (2)  Horticultural therapist (1) |
|  | Education session | 30 | Gardening activities |  |
|  | Exercise session | 40 | Indoor exercise for the elderly |  |
|  | Closing | 10 | Wrap-up and next week’s schedule announcement |  |
| 8 | Introduction | 10 | Program introduction and schedule overview | CHN (2) |
|  | Exercise session | 40 | Indoor exercise for the elderly |  |
|  | Post test | 30 | Health status assessment and satisfaction survey |  |
|  | Closing | 10 | Wrap-up |  |
| Note. CHN: community health nurse; community health nurses assigned the following unique titles to the educational program to encourage participants' interest (used from weeks 2 to 7): Active Brain 1, 2, Healthy Life at 100, Boost Your Health with Easy Tips, Back to Childhood Adventures, and I Am a Gardener. | | | | |

**Supplementary Table 3.** Description of exercise movements

| **Description** |
| --- |
| Warm up |
| Proper Walking Posture Education  ① Stand straight, looking forward.  ② March in place, lifting knees.  ③ Breathe slowly, swinging both arms back and forth naturally as if walking.  ④ 90 seconds/1 set |
| Standing Shoulder Stretch  ① Stand straight with feet shoulder-width apart.  ② Place left hand on right shoulder.  ③ Use right arm to pull left elbow towards chest.  ④ Feel the stretch in shoulder muscles while performing.  ⑤ 10~30 seconds/1 set |
| Standing Side Stretch  ① Stand straight with feet shoulder-width apart.  ② Raise one hand above head, place other hand on chair.  ③ Bend torso sideways, moving raised hand to opposite side.  ④ 10~30 seconds/1 set |
| Main exercise |
| Seated Water Bottle Bicep Curls  ① Sit upright on a chair without armrests, not leaning on backrest.  ② Hold water bottle with palms facing up, bend elbows to lift bottle.  ③ Pause for 2 seconds at the top, then slowly lower.  ④ 8~15 repetitions/3 sets |
| Seated Water Bottle Lateral Raises  ① Sit upright on a chair without armrests, not leaning on backrest.  ② Hold water bottles with palms facing down, raise arms to shoulder height.  ③ Pause for 1 second, then slowly lower.  ④ 8~15 repetitions/3 sets |
| Seated Water Bottle Tricep Extensions  ① Sit upright on a chair without armrests, not leaning on backrest.  ② Position water bottle behind head.  ③ Keep elbows fixed, extend arms upward.  ④ 8~15 repetitions/3 sets |
| Seated Water Bottle Front Raises  ① Sit upright on a chair without armrests, not leaning on backrest.  ② Keep feet flat on the floor.  ③ Hold water bottles with palms facing inward.  ④ Raise arms forward while turning palms upward.  ⑤ Pause for 1 second, then slowly lower.  ⑥ 8~15 repetitions/3 sets |
| Sit-to-stand from chair  ① Sit upright on a chair without armrests, feet shoulder-width apart.  ② Place hands on thighs, position feet slightly ahead of knees.  ③ Stand up on "one," sit down on "two."  ④ 8~12 repetitions/1 set |
| Standing Knee Lifts  ① Stand behind a chair and hold it with both hands.  ② On "one," lift and lower the right leg. On "two," lift and lower the left leg.  ③ 8~12 repetitions/1 set |
| Standing Knee Bends  ① Stand behind a chair and hold it with both hands.  ② On "one," lift the right foot backwards and lower it. On "two," lift the left foot backwards and lower it.  ③ 8~12 repetitions/1 set |
| Standing Side Leg Raises  ① Stand behind a chair and hold it with both hands.  ② On "one," lift the right leg to the side and lower it. On "two," lift the left leg to the side and lower it.  ③ 8~12 repetitions/1 set |
| Wrap up |
| Marching in Place  ① Stand straight, looking forward.  ② Perform marching in place, lifting knees.  ③ Breathe slowly, swinging both arms back and forth naturally as if walking.  ④ 90 seconds/1 set |
| 6-Direction Neck Stretch  ① Stand straight and clasp hands behind head.  ② Gently pull your hands downward.  ③ Lift head to look up, then clasp hands and gently lift chin.  ④ Facing forward, tilt head to the left. Use left hand to gently pull the right side of head (repeat on the opposite side).  ⑤ Facing forward, tilt head diagonally to the left. Use left hand to gently pull the opposite side of head (repeat on opposite side).  ⑥ 15 seconds for each direction/1 set |
| Shoulder Stretch  ① Extend left arm forward, then move it towards right shoulder.  ② In this position, use right forearm to pull left arm towards chest. (Repeat on opposite side)  ③ 15~20 seconds/1 set |
| Chest Stretch  ① Lower both hands, bend elbows, and spread arms to the sides.  ② Bring shoulder blades together and push chest forward.  ③ Conversely, raise both hands up, bring shoulder blades together, and push chest forward.  ④ 15 seconds/1 set |
| Back and Rear Shoulder Stretch  ① Sit in a chair, interlace fingers, and extend arms forward.  ② Round back and push it backwards.  ③ Extend hands forward as if pushing.  ④ 15~20 seconds/1 set |
| Torso Rotation  ① Sit in a chair and twist body to the left.  ② Hold the chair's armrests or backrest with both hands.  ③ Keep pelvis as stable as possible and twist a bit more. (Repeat on opposite side)  ④ 15~20 seconds/1 set |
| Seated Toe Touch  ① While seated, extend one leg forward and pull foot towards your body.  ② Lean forward and grasp extended foot with both hands.  ③ Gradually lean upper body forward. (Repeat on opposite side)  ④ 15~20 seconds/1 set |
| Seated Knee to Chest  ① While seated, bend one knee and place it on top of the opposite knee.  ② Hold the bottom of the bent leg with both hands and slowly pull upwards. (Repeat on opposite side)  ③ 15~20 seconds/1 set |
| Calf Stretch  ① Stand near a chair or wall for balance.  ② Pull foot towards body.  ③ Rest the front part of foot against the chair or wall to stretch. (Repeat on opposite side)  ④ 15~20 seconds/1 set |
| **Note.** The exercise movement images and descriptions used for the intervention in this study are described from the exercise program manual provided on the Korea Health Promotion Institute website (<https://www.khepi.or.kr/>) |

**Supplementary Table 4.** Baseline characteristics of completers and dropouts

|  |  | **n (%) or M ± SD** | |  |  |
| --- | --- | --- | --- | --- | --- |
| **Variables** |  | **Completers (n=92)** | **Dropouts (n=7)** | **t or χ²** | **p-value** |
| *Sociodemographic factors* | |  |  |  |  |
| Gender | Male | 10 (10.87) | 0 (0.00) | 0.85 | 0.358 |
|  | Female | 82 (89.13) | 7 (100.00) |  |  |
| Age (continuous) |  | 76.98±5.43 | 78.29±5.91 | -0.61 | 0.543 |
| Age (categorical) | 65-74 | 29 (31.52) | 1 (14.29) | 0.92 | 0.339 |
|  | ≧85 | 63 (68.48) | 6 (85.71) |  |  |
| Spouse cohabitation status | No | 61 (66.30) | 6 (85.71) | 1.12 | 0.290 |
|  | Yes | 31 (33.70) | 1 (14.29) |  |  |
| Live alone | No | 30 (32.61) | 2 (28.57) | 0.05 | 0.826 |
|  | Yes | 62 (67.39) | 5 (71.43) |  |  |
| Education level (continuous) |  | 6.76±3.90 | 6.43±3.64 | 0.22 | 0.828 |
| Education level (categorical) | ≦6 years | 54 (58.70) | 5 (71.43) | 0.44 | 0.508 |
|  | >6 years | 38 (41.30) | 2 (28.57) |  |  |
| Current working status | No | 82 (89.13) | 6 (85.71) | 0.08 | 0.782 |
|  | Yes | 10 (10.87) | 1 (14.29) |  |  |
| *Metabolic health indicators* | |  |  |  |  |
| BMI |  | 23.09±3.45 | 24.29±3.40 | -0.88 | 0.379 |
|  | <25kg/m2) | 66 (71.74) | 4 (57.14) | 0.67 | 0.413 |
|  | ≧25kg/m2) | 26 (28.26) | 3 (42.86) |  |  |
| SBP (mmHg) |  | 129.21±20.87 | 141.00±20.80 | -1.44 | 0.153 |
|  | <130 | 48 (52.17) | 1 (14.29) | 3.74 | 0.053 |
|  | ≧130 | 44 (47.83) | 6 (85.71) |  |  |
| DBP (mmHg) |  | 73.01±11.69 | 81.43±16.11 | -1.79 | 0.077 |
|  | <80 | 67 (72.83) | 4 (57.14) | 0.79 | 0.374 |
|  | ≧80 | 25 (27.17) | 3 (42.86) |  |  |
| BST (mg/dl) |  | 159.72±58.30 | 130.29±26.38 | 1.32 | 0.190 |
|  | <140 | 40 (43.48) | 4 (57.14) | 0.49 | 0.483 |
|  | ≧140 | 52 (56.52) | 3 (42.86) |  |  |
| *Functional capacity* |  |  |  |  |  |
| Grip strength (kg) |  | 19.91±4.92 | 18.60±2.84 | 0.69 | 0.490 |
| Low grip strength | Yes | 40 (43.48) | 4 (57.14) | 0.49 | 0.483 |
|  | No | 52 (56.52) | 3 (42.86) |  |  |
| Frailty |  | 8.32±2.85 | 8.86±2.12 | -0.49 | 0.624 |
|  | Pre-frail (4-12) | 86 (93.48) | 6 (85.71) | 0.60 | 0.440 |
|  | Frail (≧13) | 6 (6.52) | 1 (14.29) |  |  |
| **Note.** Means (M) ± standard deviation (SD) for continuous variables and frequencies (n) with percentages (%) for categorical variables are presented. Low grip strength is defined as <28 kg for males and <18 kg for females. | | | | | |

**Supplementary Table 5.** Comparison of pre- and post-intervention outcomes using bootstrapped paired t-test and Wilcoxon signed-rank test (N=92)

| **Variables** | **M ± SD** | | **Paired t-test (p-value)** | **Bootstrapped 95% CI** | **Wilcoxon Z** | **Wilcoxon p-value** |
| --- | --- | --- | --- | --- | --- | --- |
|  | **Pre-intervention** | **Post-intervention** |  |  |  |  |
| BMI (kg/m2) | 23.09±3.45 | 23.16±3.41 | -1.02  (0.311) | [-0.19, 0.06] | -1.25 | 0.210 |
| SBP (mmHg) | 129.21±20.87 | 127.09±17.22 | 1.24  (0.219) | [-1.25, 5.34] | -1.20 | 0.229 |
| DBP (mmHg) | 73.01±11.69 | 72.54±10.76 | 0.43  (0.665) | [-1.74, 2.49] | -.63 | 0.531 |
| BST (mg/dl) | 159.72±58.3 | 139.28±50.3 | 4.54  (<0.001) | [11.39, 29.30] | -4.29 | <0.001 |
| TUG TEST (sec) | 9.65±3.45 | 8.00±3.07 | 7.47  (<0.001) | [1.24, 2.07] | -6.52 | <0.001 |
| Grip strength (kg) | 19.91±4.92 | 21.05±5.18 | -4.22  (<0.001) | [-1.66, -0.62] | -4.03 | <0.001 |
| Frailty | 8.32±2.85 | 6.72±2.67 | 7.17  (<0.001) | [1.35, 2.26] | -6.17 | <0.001 |
| **Note.** Means (M) ± standard deviation (SD) for continuous variables is presented. | | | | | | |

**Supplementary Table 6.** Comparison of specific frailty domains in pre- and post-intervention among older adults (N=92)

|  |  |  | **M±SD** | |  |  |  |
| --- | --- | --- | --- | --- | --- | --- | --- |
| **Sub-domain** | **Score range** |  | **Pre-intervention** | **Post-intervention** | **t** | ***p*-value / Cohen’s d** | **95%CI** |
| ADL | 0–5 |  | 0.55±0.79 | 0.46±0.73 | 2.00 | 0.049 / 0.21 | [0.00, 0.20] |
| Mobility | 0–3 |  | 1.12±0.94 | 0.84±0.83 | 3.50 | 0.001 / 0.36 | [0.12, 0.44] |
| Nutrition | 0–2 |  | 0.15±0.42 | 0.13±0.34 | 0.71 | 0.483 / 0.07 | [-0.04, 0.08] |
| Oral function | 0–3 |  | 0.57±0.83 | 0.51±0.75 | 0.76 | 0.449 / 0.08 | [-0.09, 0.20] |
| Social health | 0–2 |  | 0.34±0.50 | 0.2±0.4 | 2.81 | 0.006 / 0.29 | [0.04, 0.24] |
| Cognitive function | 0–3 |  | 0.36±0.62 | 0.35±0.56 | 0.17 | 0.863 / 0.02 | [-0.11, 0.14] |
| Depressive symptoms | 0–5 |  | 0.87±1.09 | 0.5±0.87 | 4.00 | <0.001 / 0.42 | [0.19, 0.55] |
| Sensory function | 0-2 |  | 0.45±0.64 | 0.18±0.39 | 4.70 | <0.001 / 0.49 | [0.15, 0.37] |
| Gait function | 0-2 |  | 1.00±1.01 | 0.59±0.92 | 4.12 | <0.001 / 0.96 | [0.21, 0.64] |
| **Note.** ADL: Activities of daily living, t-values for continuous variables are presented, with means (M) ± standard deviation (SD) for continuous variables. | | | | | | |  |
